# Metatarsophalangeal Joint Reconstruction Using Talar Osteochondral Allograft following a Failed Dorsal Cheilectomy

**DOI:** 10.1155/2022/6359108

**Published:** 2022-09-19

**Authors:** Alexandria J. Lichtl, Kelly L. Vittetoe, Connie P. Friedman, Hardik P. Parikh, Christopher S. Lee

**Affiliations:** ^1^NYU Grossman School of Medicine, USA; ^2^Vanderbilt University Medical Center, USA; ^3^Ventura County Medical Center, USA; ^4^UCLA Spine Center, USA; ^5^Stetson Lee Orthopaedics and Sports Medicine, 191 S. Buena Vista St., Suite, #470 Burbank, CA 91505, USA

## Abstract

Dorsal cheilectomy is often used as a first-line surgical treatment for hallux rigidus; however, revision surgery is needed in nearly 9% of cases. One option for revision surgery is interpositional arthroplasty, which is designed to preserve joint motion and is favorable in young, active populations. This case discusses a young female patient with persistent, painful hallux rigidus and a large osteochondral defect despite prior dorsal cheilectomy. We performed an interpositional arthroplasty of the first metatarsophalangeal joint using an osteochondral allograft from the talus. At three-year follow-up, she had greatly improved function and was able to run without pain. To our knowledge, this is the first documented use of an osteochondral allograft from the talus in conjunction with metatarsophalangeal joint interpositional arthroplasty for treatment of hallux rigidus and a severe osteochondral defect. This technique introduces osseous subchondral scaffolding as well as mature hyaline cartilage into an osteochondral lesion, thereby reestablishing proper joint architecture and congruent articulation and ultimately improving range of motion and reducing pain. We present this technique as an experimental treatment option for restoring both the integrity and function of the metatarsophalangeal joint following trauma, osteochondritis dissecans, or prior operative failure in patients who wish to delay metatarsophalangeal joint fusion.

## 1. Introduction

Hallux rigidus is a common source of foot pain that is caused by osteoarthritis of the first metatarsophalangeal (MTP) joint. Symptomatic hallux rigidus is estimated to be present in nearly 8% of adults and is the most common degenerative joint disease of the foot [1, 2]. Dorsal cheilectomy has been proposed as a first-line surgical treatment for this condition; however, a systematic review indicated that 8.8% of isolated cheilectomies required revision surgery [3]. Decompression osteotomy is also commonly used to treat hallux rigidus and results in beneficial long-term outcomes with a lower rate of revision than dorsal cheilectomy; however, decompression osteotomy is typically more difficult to perform and poses more risk to the patient [4, 5]. Arthrodesis is an option for hallux rigidus revision surgery that has a high success rate in pain relief, but is not ideal in younger populations due to loss of joint motion and activity limitations [6]. For similar reasons, implant arthroplasty and resection arthroplasty are not favorable for young, active patients [7]. Interpositional arthroplasty, which uses a biologic as a spacer in the MTP joint, was developed in response to these limitations. In prior studies, interpositional arthroplasty of the MTP joint has been performed with Silastic material, regenerative tissue matrix, and bioresorbable implants, as well as autograft transfer from the medial and plantar aspect of the ipsilateral talar head [8–12]. Since interpositional arthroplasty is designed to preserve more bone and joint motion, thereby improving function, it has great potential to improve outcomes in active patients [13].

Osteochondral lesion of the first MTP joint in hallux rigidus requires additional consideration. Current arthroscopic treatments of osteochondral defects of the first MTP joint include sesamoid excision, synovectomy, debridement, and partial cheilectomy, which can be painful procedures [14]. Microhole drill and microfracture techniques are additional options; however, in general, marrow-inducing approaches have not been shown to sustain long-term benefits due to the inferior biomechanical properties of the fibrocartilage repair tissue [14, 15]. Osteochondral autograft implantation is also used to treat osteochondral lesions, but subsequent pressure on the donor site can increase morbidity [15, 16]. Autologous chondrocyte implantation (ACI) poses less risk of donor site morbidity, but requires two sequential procedures of harvesting and implantation, which can be time-consuming and inconvenient for the patient [17, 18].

Osteochondral allograft (OCA) transplantation is regarded as a salvage procedure for large osteochondral lesions, including patellofemoral and talar that are too large for other reparative or restorative procedures [15, 19]. Recent studies have examined the use of fresh OCA to correct hallux rigidus and have found this approach to be a viable option [20, 21]. However, these cases have focused on treatment of either early hallux rigidus or end-stage progressive arthritis, so little is known about the efficacy of OCA for the treatment of large osteochondral defects of the metatarsal head due to causes such as trauma, osteochondritis dissecans, or prior operative failure.

In this case, a young patient presented with persistent hallux rigidus and 50% surface area loss of the metatarsal head following a failed dorsal cheilectomy. Due to the large size of the osteochondral defect and the patient's desire for long-term joint mobility, we felt OCA and interpositional arthroplasty were the best approach. We used fresh OCA to restore anatomical joint architecture and articulation in the MTP joint of the great toe. We believe this is the first documented case in which OCA of the talus was used in conjunction with MTP joint interpositional arthroplasty to treat hallux rigidus and a large osteochondral lesion following failure of first-line treatment.

## 2. Case Report

A 24-year-old female was under our care for three years. At presentation, she had no past medical history or pertinent family medical history and was not taking any medications. She was referred to our office for treatment of her right great toe, at which point she was 11 months status post a right great toe dorsal cheilectomy performed by an outside physician. She reported making an incomplete recovery with regard to pain and functionality despite three cortisone injections and postoperative physical therapy. She presented with pain at the MTP joint of her right great toe associated with swelling, weakness, limited range of motion, and instability.

### 2.1. Treatment

**Figure 1:**
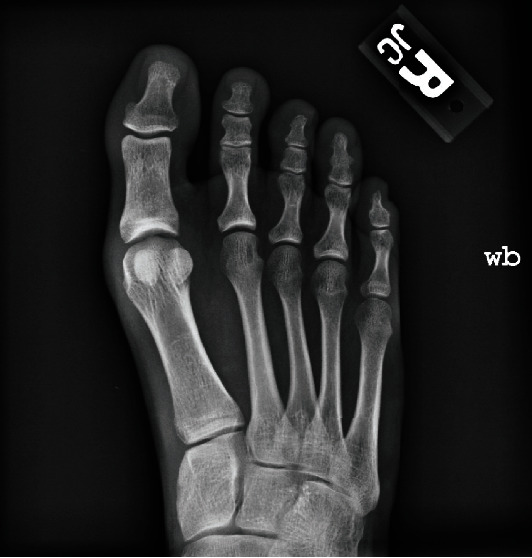
Preoperative right foot anteroposterior radiograph taken at the time of presentation.

**Figure 2:**
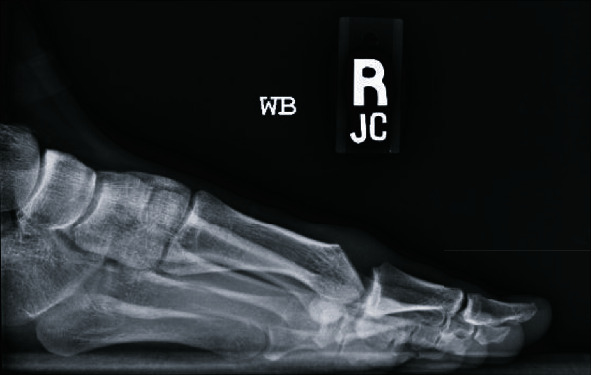
Preoperative right foot lateral radiograph taken at the time of presentation showing prior dorsal cheilectomy.

Weightbearing anteroposterior, lateral, and oblique radiographs of the patient's right foot taken at her initial visit showed evidence of previous dorsal cheilectomy with no fractures or dislocations (Figures 1 and 2).

**Figure 3:**
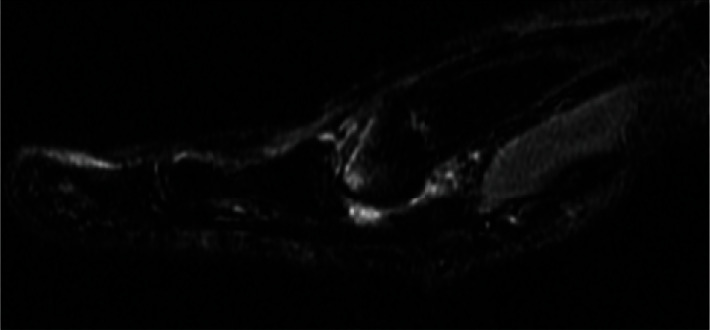
Preoperative right foot MRI performed 14-month status postdorsal cheilectomy demonstrating 50% loss of the first metatarsal head.

Her initial clinical examination was concerning for capsulitis versus osteomyelitis, due to persistent postoperative edema and erythema. An ultrasound-guided aspiration of her right great toe was attempted at her initial visit, but no fluid was aspirated. She was advised to continue physical therapy and wear a postoperative shoe. Following three months of physical therapy, the patient's right great toe pain and symptoms had improved only slightly. Due to her persistent pain and reduced function, T1, T2, and STIR magnetic resonance imaging (MRI) sequences of the right foot were obtained in the sagittal, axial, and true and oblique coronal planes. The imaging, which was obtained 14 months status post dorsal cheilectomy, showed evidence of prior first metatarsal head cheilectomy/osteotomy with contour deformity dorsally and persistent marrow edema thought to be related to postoperative changes. Also noted was 50% surface area loss of the first metatarsal head (Figure 3).

Following review of the MRI, operative treatment described as an allograft spacer versus a fresh allograft transplant was discussed, and the patient ultimately decided to proceed with surgery. Given the surface area loss of the metatarsal head observed on MRI, we believed that OCA transplantation would best restore congruence of the metatarsal head and correct the posterior subluxation of the first MTP joint.

### 2.2. Surgical Technique

Examination under anesthesia of the first MTP joint demonstrated the patient's range of motion to be limited to 30 degrees of dorsiflexion and 40 degrees of plantarflexion. The contralateral healthy side demonstrated 70 degrees of dorsiflexion and 90 degrees of plantarflexion.

**Figure 4:**
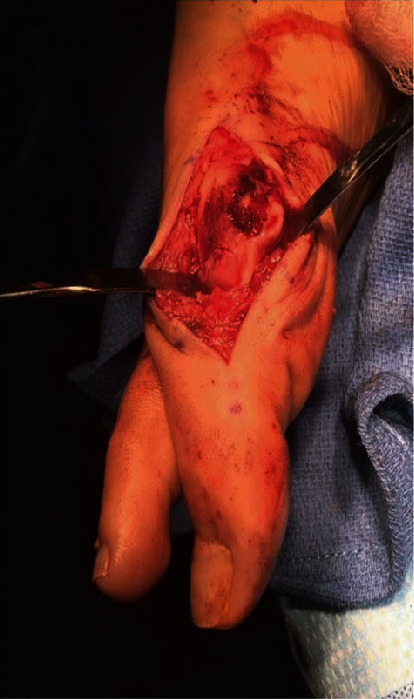
Dorsomedial incision of the first MTP joint shows Outerbridge classification grade IV chondromalacia with 50% of metatarsal head cartilage missing.

A standard incision was made over the dorsomedial aspect of the first MTP joint (Figure 4). The dorsal nerve was identified and retracted posteriorly. This was followed by a capsulotomy, exposing the MTP joint. Severe adhesions were elevated anteriorly, which were scarred to the exposed medullary bone from the previous cheilectomy. A large osteophyte at the medial aspect of the first metatarsal head that had caused red, swollen, and inflamed skin was identified and excised with a small saw blade. The resection was flushed with the remaining metatarsal neck. A rongeur and a hand rasp were used to smooth the metatarsal, thereby completing the open bunionectomy.

Postbunionectomy, the hallux rigidus correction with implant was performed. Dorsiflexion of the first MTP joint demonstrated articulation of the dorsal aspect of the proximal phalanx with exposed medullary bone on the metatarsal head. This articulation might have deteriorated much of the cartilage to the point where the dorsal half of the metatarsal head was absent, including the articular cartilage, yielding bone-on-bone articulation.

A saw blade was used to gently freshen the soft and osteoporotic bone on the dorsal metatarsal head. Approximately 1 millimeter (mm) was resected from the metatarsal head to obtain fresh medullary bone for healing surface. At least 50% of the dorsal osteochondral aspect of the metatarsal head had been abraded through dorsiflexion, as measured intraoperatively and shown on the preoperative MRI. Cartilage from the proximal phalanx was seen articulating on the bone of the distal metatarsal with Outerbridge classification grade IV chondromalacia on the dorsal aspect.

To address the large osteochondral lesion, a fresh talus allograft was matched with the metatarsal head to create a graft that measured 10 mm in height by 15 mm in length by 15 mm in width. The aspect of the talus that articulates with the navicular matched the metatarsal head exactly. The portion of talonavicular joint cartilage that matched the contour of the metatarsal head measuring 10 × 15 × 15 mm was resected from the talus. The OCA was matched to the metatarsal head for a flush fit and the proximal aspect of the graft was further contoured to match the angle of the abraded metatarsal head. Finally, the graft was positioned on the deficient metatarsal head to test the graft's articulation with the proximal phalanx. Intraoperative tests showed no evidence of proximal phalanx bone-on-bone articulation in either dorsiflexion or plantarflexion with excellent dorsiflexion and cartilage-on-cartilage articulation. Demonstration of the graft with the metatarsal head also showed restored stability, as compared to preoperative MRI findings, which originally revealed joint instability with volar subluxation of the metatarsal head and dorsal subluxation of the proximal phalanx due to the lack of a dorsal metatarsal head (Figure 3).

**Figure 5:**
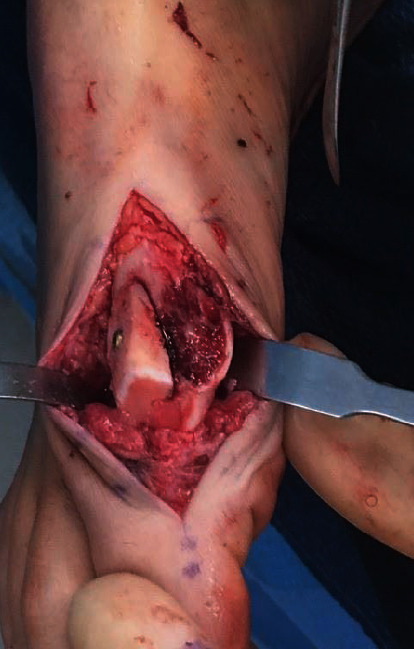
Fresh talonavicular joint cartilage implant increased dorsiflexion and plantarflexion by 20 degrees during the operation.

The metatarsal head was further contoured and affixed to the first metatarsal using a 2.5 × 18 mm screw implant® (Stryker, Kalamazoo, MI). The headless screw, inserted from dorsal to volar, avoided the articular cartilage and provided excellent finishing and compression. Biplanar C-arm fluoroscopy views ensured the appropriate screw length without plantar exposure to prevent the patient from stepping directly on the screw. We then used a pineapple burr to shape the graft to match the contour of the metatarsal head. The dorsal aspect of the graft was secured to the remaining metatarsal with excellent stability (Figure 5). Following the hallux rigidus correction with the implant, dorsiflexion and plantarflexion increased to about 50 and 60 degrees, respectively. This completed the hallux rigidus correction with OCA implantation on the metatarsal head.

To further correct the bunion deformity, the medial capsule was over sewn with a No. 1 Vicryl in a horizontal mattress stitch. Subcutaneous tissue was closed with a 3-0 Vicryl in inverted fashion, and the skin was closed with a 4-0 nylon interrupted horizontal mattress fashion. Xeroform was applied, and a gauze was placed between the first and second toes to keep the great toe in a varus position. Finally, a posterior plaster splint was applied in 90 degrees of ankle dorsiflexion.

### 2.3. Outcome and Follow-Up

**Figure 6:**
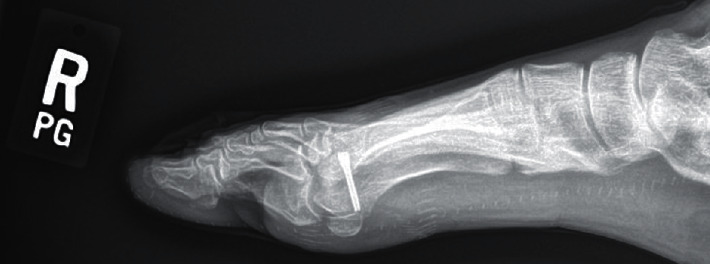
Postoperative lateral radiograph of the right foot demonstrating hallux rigidus correction with OCA implantation on the metatarsal head.

**Figure 7:**
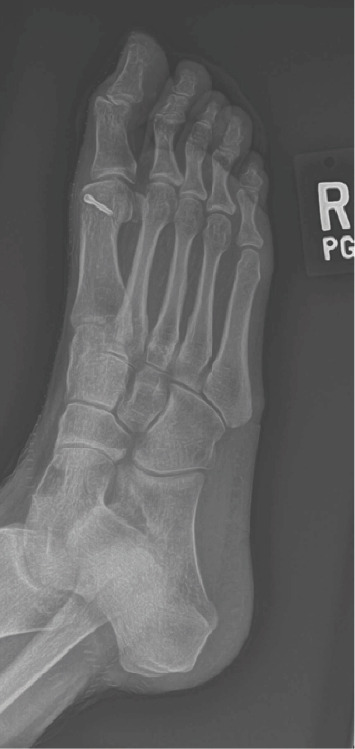
Postoperative oblique radiograph of the right foot demonstrating hallux rigidus correction with OCA implantation on the metatarsal head.

The patient returned to our office six days postoperatively with improved range of motion and reduced pain. The patient was advised to weight bear as tolerated and begin a formal postoperative physical therapy program with an emphasis on foot and ankle strengthening and proprioception. She returned to our office five weeks thereafter and reported continued improvement of range of motion. She was advised to discontinue use of crutches and begin walking with a cast shoe. Postoperative radiographs taken at three month follow-up revealed correction of the hallux rigidus and osteochondral lesion with no fractures or dislocations (Figures 6 and 7). She returned to the clinic four months following surgery and reported improved pain and function of the right first MTP joint. Compared to preoperative range of motion, her postoperative dorsiflexion increased by 15 degrees from 30 to 45 degrees and plantarflexion increased by 25 degrees from 45 to 70 degrees. However, at one year following surgery, the patient reported worsening pain and difficulty walking. Her symptoms were suspected to be related to painful hardware, and the 2.5 mm headless screw implant was removed. Over the next nine months, she received additional treatment including a cortisone injection, physical therapy, and home exercises with improvement of symptoms.

**Figure 8:**
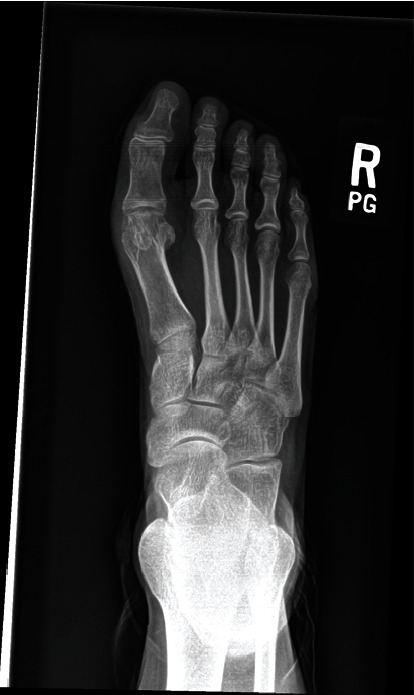
Anteroposterior radiograph of the right foot taken three years post-OCA implantation with complete resolution of hallux rigidus.

**Figure 9:**
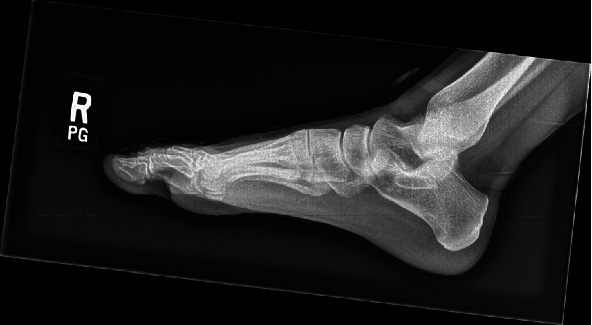
Lateral radiograph of the right foot taken three years post-OCA implantation with complete resolution of hallux rigidus.

**Figure 10:**
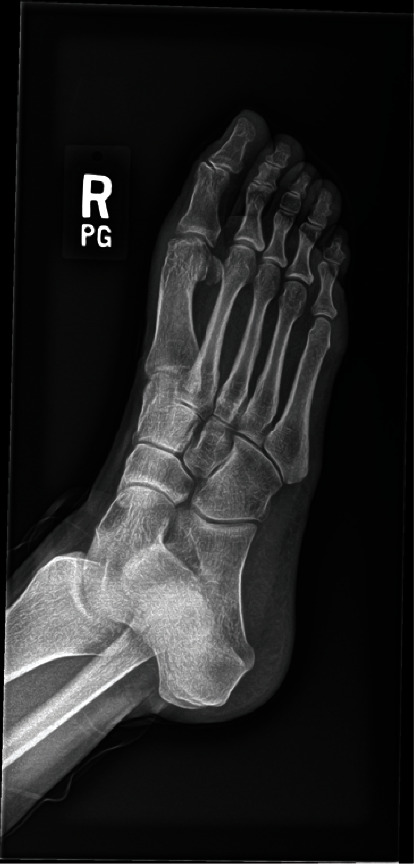
Oblique radiograph of the right foot taken three years post-OCA implantation with complete resolution of hallux rigidus.

At one year and nine months postoperatively, a right foot MRI without contrast was performed to evaluate for graft integrity. The MRI revealed that there was no full-thickness defect or chondral delamination and no evidence of tendon tear or tenosynovitis. At two and a half year follow-up, the patient returned and reported great improvement to her symptoms. She denied swelling, locking/catching, or instability, and physical examination showed excellent strength and range of motion. She was also able to ambulate with ease and was pain-free. At three-year follow-up, the patient was able to run up to two miles without pain and had begun weightlifting. She reported that her right foot symptoms had entirely resolved. Radiographs taken at that time revealed complete resolution of hallux rigidus (Figures 8–10).

## 3. Discussion

Although osteochondral lesions of the MTP joint are not as common as knee or ankle joint osteochondral lesions, they pose a risk for severe pain and limited activity due to their weightbearing character. Osteochondral lesions of the MTP joint may present with severe mechanical symptoms, such as grinding, as well as disrupted flexion of the great toe, causing an inability to walk [9]. There are few studies that describe treatment of large osteochondral lesions of the MTP joint; therefore, the optimal form of treatment for this condition remains unknown. Literature regarding OCA treatment of osteochondral lesions in other weightbearing joints exhibited its promise for our particular case [22, 23]. OCA has been used as a salvage procedure for patients who are poor candidates for arthroplasty procedures and sustain large osteochondral lesions [15].

This case study presents OCA as a feasible option for treating hallux rigidus with a severe osteochondral lesion following a great toe cheilectomy with incomplete recovery. By using a portion of the talus that articulates with the navicular as the OCA, we were able to restore congruent articulation, improve range of motion, and reduce pain. The restorative approach of OCA contrasts with reparative approaches that aim to relieve symptoms but do not necessarily restore articular cartilage architecture. Restorative approaches achieve complete reconstruction of the microarchitecture of articular cartilage to restore the biomechanical and physiological properties of the affected joint [24]. The technique used in this case, modeled after OCA treatments for osteochondral defects in the knee, introduces both subchondral bone and mature hyaline cartilage to a previously damaged articular surface. Hyaline cartilage—as compared to fibrocartilage generated through reparative techniques like microfracture—is ideal for transplantation because it is avascular, aneural tissue that is protected from host immune detection, as its chondrocytes are embedded within the cellular matrix [25].

Currently, OCA is the only biomimetic technique that restores mature, architecturally appropriate hyaline cartilage to an articular surface lesion [25]. Prior case studies investigating the efficacy of OCA in the patellofemoral joint have shown encouraging graft survivorship and patient satisfaction ratings [18, 24]. Increased accessibility of fresh donor tissue coupled with rising patient and physician demand for technological advancements in articular cartilage repair has fueled the growth of biologic resurfacing as an alternative to joint replacement [17, 25]. In light of these advances as well as the advantages of OCA described above, this report offers OCA as a novel methodology for operative treatment of hallux rigidus following a dorsal cheilectomy with incomplete recovery.

In this case, OCA with concurrent interpositional arthroplasty has been shown to be an effective method for restoring both the integrity and function of the MTP joint and has the potential to be used following trauma, osteochondritis dissecans, or first-line operative failure. This procedure had an extended recovery, as the patient did not reach maximum function until more than two years after surgery and required additional treatment in the form of cortisone injections and physical therapy. Therefore, patients and clinicians should be prepared for a long-term recovery if they utilize this surgical technique in the future. Given that hallux rigidus is a debilitating condition that affects a large number of adults, it is important to better understand possible treatment approaches, especially in the case that first-line operative treatment fails. This restorative technique introduces osseous subchondral scaffolding as well as mature hyaline cartilage into an osteochondral lesion, thereby reestablishing proper joint architecture and congruent articulation following a failed cheilectomy. Since this is a novel approach to treating an established pathological condition, additional cases should be described to determine the practicality and value of this procedure on a broader basis.

## Data Availability

Data can be accessed by contacting Dr. Christopher Lee at chrisleemd08@gmail.com or 818-848-3030.

## Additional Points

The patient in the case study paid for her medical services through insurance and self-pay.

## References

[1] Roddy E., Thomas M. J., Marshall M. (2015). The population prevalence of symptomatic radiographic foot osteoarthritis in community-dwelling older adults: cross-sectional findings from the clinical assessment study of the foot. *Annals of the Rheumatic Diseases*.

[2] Ho B., Baumhauer J. (2017). Hallux rigidus. *EFORT Open Reviews*.

[3] Roukis T. S. (2010). The need for surgical revision after isolated cheilectomy for hallux rigidus: a systematic review. *The Journal of Foot and Ankle Surgery*.

[4] Cullen B., Stern A. L., Weinraub G. (2017). Rate of revision after cheilectomy versus decompression osteotomy in early- stage hallux rigidus. *The Journal of Foot and Ankle Surgery*.

[5] Slullitel G., López V., Calvi J. P., D’Ambrosi R., Usuelli F. G. (2020). Youngswick osteotomy for treatment of moderate hallux rigidus: thirteen years without arthrodesis. *The Journal of Foot and Ankle Surgery*.

[6] Erdil M., Elmadag N. M., Polat G. (2013). Comparison of arthrodesis, resurfacing hemiarthroplasty, and total joint replacement in the treatment of advanced hallux rigidus. *The Journal of Foot and Ankle Surgery*.

[7] Berlet G. C., Hyer C. F., Lee T. H., Philbin T. M., Hartman J. F., Wright M. L. (2008). Interpositional arthroplasty of the first MTP joint using a regenerative tissue matrix for the treatment of advanced hallux rigidus. *Foot & Ankle International*.

[8] DelaCruz E. L., Johnson A. R., Clair B. L. (2011). First metatarsophalangeal joint interpositional arthroplasty using a meniscus allograft for the treatment of advanced hallux rigidus: surgical technique and short-term results. *Foot & Ankle Specialist*.

[9] McNeil D. S., Baumhauer J. F., Glazebrook M. A. (2013). Evidence-based analysis of the efficacy for operative treatment of hallux rigidus. *Foot & Ankle International*.

[10] Sherman S. L., Garrity J., Bauer K., Cook J., Stannard J., Bugbee W. (2014). Fresh osteochondral allograft transplantation for the knee: current concepts. *The Journal of the American Academy of Orthopaedic Surgeons*.

[11] Hyer C. F., Granata J. D., Berlet G. C., Lee T. H. (2012). Interpositional arthroplasty of the first metatarsophalangeal joint using a regenerative tissue matrix for the treatment of advanced hallux rigidus: 5-year case series follow-up. *Foot & Ankle Specialist*.

[12] Partio N., Ponkilainen V. T., Rinkinen V. (2021). Interpositional arthroplasty of the first metatarsophalangeal joint with bioresorbable pldla implant in the treatment of hallux rigidus and arthritic hallux valgus: a 9-year case series follow-up. *Scandinavian Journal of Surgery*.

[13] Brage M. E., Ball S. T. (2002). Surgical options for salvage of end-stage hallux rigidus. *Foot and Ankle Clinics*.

[14] Kuyucu E., Mutlu H., Mutlu S., Gülenç B., Erdil M. (2017). Arthroscopic treatment of focal osteochondral lesions of the first metatarsophalangeal joint. *Journal of Orthopaedic Surgery and Research*.

[15] Alford J. W., Cole B. J. (2005). Cartilage restoration, part 1. *The American Journal of Sports Medicine*.

[16] Kim Y. S., Park E. H., Lee H. J., Koh Y. G., Lee J. W. (2012). Clinical comparison of the osteochondral autograft transfer system and subchondral drilling in osteochondral defects of the first metatarsal head. *The American Journal of Sports Medicine*.

[17] Alford J. W., Cole B. J. (2005). Cartilage restoration, part 2: techniques, outcomes, and future directions. *The American Journal of Sports Medicine*.

[18] Gracitelli G. C., Meric G., Pulido P. A., Gortz S., De Young A. J., Bugbee W. D. (2015). Fresh osteochondral allograft transplantation for isolated patellar cartilage injury. *The American Journal of Sports Medicine*.

[19] VanTienderen R. J., Dunn J. C., Kusnezov N., Orr J. D. (2017). Osteochondral allograft transfer for treatment of osteochondral lesions of the talus: a systematic review. *Arthroscopy*.

[20] Giannini S., Buda R., Ruffilli A., Pagliazzi G., Vannini F. (2013). Bipolar fresh osteochondral allograft for the treatment of hallux rigidus. *Foot & Ankle International*.

[21] Hollawell S., Moen R., Coleman M., Carson M. (2021). Osteochondral fresh allograft transfer to address osteochondral defect of the first metatarsal head in early hallux limitus. *The Journal of Foot and Ankle Surgery*.

[22] Emmerson B. C., Görtz S., Jamali A. A., Chung C., Amiel D., Bugbee W. D. (2007). Fresh osteochondral allografting in the treatment of osteochondritis dissecans of the femoral condyle. *The American Journal of Sports Medicine*.

[23] Levy Y. D., Görtz S., Pulido P. A., McCauley J. C., Bugbee W. D. (2013). Do fresh osteochondral allografts successfully treat femoral condyle lesions?. *Clinical Orthopaedics and Related Research*.

[24] Cameron J. I., Pulido P. A., McCauley J. C., Bugbee W. D. (2016). Osteochondral allograft transplantation of the femoral trochlea. *The American Journal of Sports Medicine*.

[25] Görtz S., Bugbee W. D. (2006). Allografts in articular cartilage repair. *Journal of Bone and Joint Surgery*.

